# Speech language pathology evaluation is associated with decreased mortality in hip fracture patients with dysphagia

**DOI:** 10.1007/s00590-025-04533-9

**Published:** 2025-10-03

**Authors:** Corinne Vennitti, Rohan Boyapati, Kathryn Riggs, Tina Daoud, Michael Hadeed

**Affiliations:** Department of Orthopaedic Surgery, University of Virginia, Charlottesville, USA

**Keywords:** Dysphagia, Hip fracture, Geriatric fracture, Mortality, Complications, Speech language pathology

## Abstract

**Purpose:**

Dysphagia is prevalent within the geriatric hip fracture population and can lead to postoperative complications such as malnutrition, aspiration, and mortality. This study evaluates the impact of speech language pathology (SLP) evaluations and dietary changes in this population and their impact on mortality.

**Methods:**

A retrospective cohort study including patients ≥ 65 years old who underwent surgical treatment for hip fracture from January 2015 to December 2020 at an academic level 1 tertiary center was completed. The rate of dysphagia and its association with mortality were noted. Outcome measurements included the rate of SLP evaluation, the rate of diet modifications, and the effect of these interventions on mortality risk in the cohort of patients with dysphagia.

**Results:**

Within the cohort, 56% (347/617) patients had dysphagia, 29% (180/617) were on a dysphagia diet, and 44% (272/617) received a SLP evaluation. Of the patients with dysphagia, 46% (161/347) were on a dysphagia diet and 77% (266/347) received a SLP evaluation. Patients with dysphagia who received an SLP evaluation had lower mortality rates (7.1%) than those who did not have an SLP evaluation (15%) (OR 0.44, CI 0.21–0.96, *p* = 0.034). Patients with dysphagia who were on a dysphagia diet had no difference in mortality rates (OR 0.82, CI 0.39–1.7, *p* = 0.60) compared with those without a dysphagia diet.

**Conclusion:**

In this cohort, SLP evaluations for patients with dysphagia significantly reduced mortality, suggesting a role for routine early identification and intervention. This suggests that SLP evaluation and treatment improves outcomes, specifically mortality, in this population.

## Introduction

Within the geriatric population, hip fractures are a common injury with an increasing prevalence and are associated with a high perioperative mortality risk—around 20% in the year after injury [1] [2–4]. Many risk factors have been evaluated to better understand the morbidity and mortality associated with geriatric hip fractures, with the goal of improving patient care [5, 6]. Despite the identification of several predictive factors, the majority of these risk factors are non-modifiable [6, 7]. Dysphagia has previously been shown to be associated with mortality, but is somewhat unique in that there are well-established treatments and mitigation strategies to minimize its clinical impact [5, 8, 9]. Early identification and treatment of dysphagia may provide an opportunity to prevent mortality, reduce healthcare burden, and accelerate the recovery for the patient.

Dysphagia is clinical diagnosis but can be assessed with video-fluoroscopic swallowing studies to determine the severity of dysphagia [10]. Accurately assessing for and diagnosing dysphagia allows for the identification of patients who are at highest risk of aspiration and malnutrition in the postoperative period [2]. These complications lead to longer or more frequent admissions, admissions to intensive care units, additional testing such as swallowing studies, speech evaluation, speech therapy, and treatment associated with aspiration pneumonia [11–13]. The cost of an admission for a patient with dysphagia was shown to be 40–44% higher than the cost of a patient without dysphagia [14, 15]. Additionally, these patients are more likely to have longer length of stays, higher rate of discharge to a facility, and higher inpatient mortality, which adds to the economic and physical burden on the patient. [14, 15]

There are multiple interventions that can be utilized both pre-operatively and post-operatively that can aid with diagnosis and management of dysphagia. Among these resources, speech language pathologists (SLP) evaluations help in early detection and management of dysphagia, significantly improving patient outcomes [16–18]. Multiple studies have been conducted in the SLP literature demonstrating strong evidence, both clinically and electromyographically, of improvement in swallowing function with speech therapy [19–22]. Speech therapy regimens, therapeutic swallowing techniques, airway protection maneuvers, and dietary texture modifications have been shown to favor early progression of oral intake and reduction in dysphagia severity, therefore reducing dysphagia associated complications [9, 10, 20].

Dysphagia diets, such as the International Dysphagia Diet Standardization Initiative (IDDSI), provide a structured approach to manage dysphagia by offering various food consistencies tailored to patient needs [23]. These diets help maintain or improve nutritional status, prevent unnecessary tube feeding, and support patient recovery [24]. Early and appropriate dietary interventions can lead to improved swallowing safety and nutritional intake, thereby reducing hospital stay and enhancing overall recovery [25].

This study aims to explore the effects of SLP evaluations and dysphagia diets on mortality in the geriatric hip fracture population. Dysphagia is associated with mortality in this population, and if this risk factor is shown to be modifiable, treatment pathways may be developed to minimize the effect of dysphagia on mortality in these patients.

## Methods

Following institutional review board approval, a retrospective cohort study was conducted at a single academic tertiary care center. The study included patients aged 65 and older who were admitted with a hip fracture diagnosis between January 2015 and December 2020. Exclusion criteria included periprosthetic fractures, pathologic fractures, fractures treated nonoperatively, or revision procedures. Patient demographic, injury, and treatment variables were collected including age, diagnosis, date of surgery, procedure, and anesthesia type. A previous study examined several variables regarding dysphagia in this cohort of patients [5]. The presence of dysphagia was recorded. The presence of a speech language pathology evaluation and whether a dysphagia diet was ordered during their hip fracture admission were recorded. Finally, mortality was recorded.

Dysphagia diagnosis was positive if there were references to dysphagic events in encounter notes, a diagnosis of dysphagia in the patient problem list, any indication of dysphagia or swallowing difficulty in the patient chart, swallow studies with a diagnosis of dysphagia, or speech language pathology evaluations for dysphagia. Postoperative mortality was defined as death occurring within 90 days after surgery.

A typical speech language pathology evaluation includes an oral motor evaluation and diet texture analysis. General and patient-specific swallowing education is provided including safety strategies. A diet is prescribed based on the evaluation, and possible further diagnostics may be recommended.

An alpha of 0.05 was used to determine statistical significance. Statistical analyses, including Chi-squared tests and univariate odds ratio analysis, were performed using Python version 3.9.12 (Beaverton, Oregon, USA).

## Results

The cohort was composed of 617 patients who underwent surgical fixation of a hip fracture. The average age was 81 years (± 8.6). Fifty-five percent of fractures were femoral neck, 40% were intertrochanteric, 4% were subtrochanteric, and 1% were not able to be classified. Thirty-three percent of patients were treated with a cephalomedullary nail, 31% with a hemiarthroplasty, 15% with a total hip arthroplasty, 14% with a sliding hip screw, and 7% with percutaneous fixation in situ. Eighty-eight percent were treated with general anesthesia and 12% with spinal anesthesia.

Within the cohort, 56% (347/617) of the patients had a diagnosis of dysphagia, 29% (180/617) were on a dysphagia diet, and 44% (272/617) received a SLP evaluation during admission (Table 1). Of the patients with a diagnosis of dysphagia, 46% (161/347) were placed on dysphagia diet and 77% (266/347) received a SLP evaluation (Table 1).

**Table 1:** Rates of SLP evaluation, dysphagia diet use, and mortality in the total cohort and dysphagia cohort

	Total cohort *n* = 617	Dysphagia cohort *n* = 347
SLP evaluation	44% (272)	77% (266)
Dysphagia diet	29% (180)	46% (161)
Mortality	6.1% (38)	8.9% (31)

**Fig. 1:**
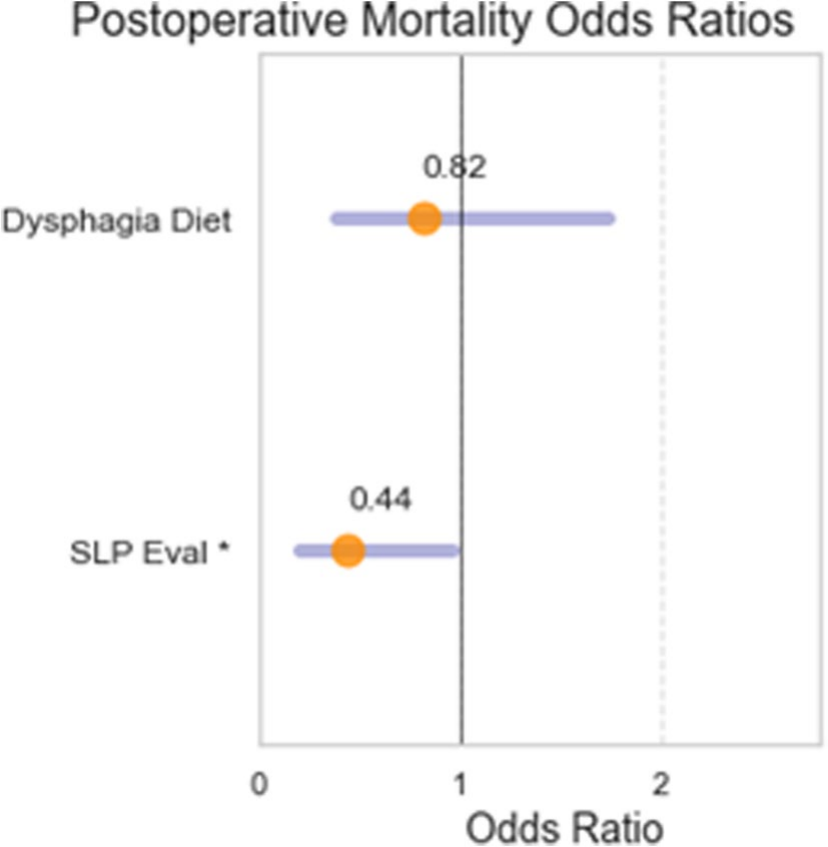
Odds ratio graph for postoperative mortality based on SLP evaluation and dysphagia diet use

Patients with dysphagia had higher postoperative mortality rates (31/347, 8.9%) than those who did not have dysphagia (7/270, 2.6%) (*p* = 0.001). Within the cohort of patients with dysphagia, patients who received an SLP evaluation had lower mortality rates (19/266, 7.1%) compared to those who did not have an SLP evaluation (12/81, 15%) (*p* = 0.034) (Table 2). Within the dysphagia cohort, there was no difference in mortality rates between patients on a dysphagia diet (13/161, 8.1%) and patients without a dysphagia diet (18/186, 9.7%) (*p* = 0.60) (Table 2). An odds ratio analysis was completed demonstrating that an SLP evaluation decreases the risk of postoperative mortality in patients with dysphagia (OR = 0.44, CI 0.21–0.96), but a dysphagia diet did not affect mortality rates (OR 0.82, CI 0.39–1.7) (Fig. 1).

**Table 2:** Rates of mortality based on SLP evaluation and dysphagia diet use in the cohort of patients with dysphagia

Dysphagia cohort, n = 347		
	Mortality	
SLP evaluation	7.1% (19)	*p* = 0.034
No SLP evaluation	15% (12)	
Dysphagia diet	8.1% (13)	*p* = 0.60
No dysphagia diet	9.7% (18)

## Discussion

Dysphagia has been previously shown to be a risk factor associated with mortality in the geriatric hip fracture population [5]. Well-established evaluation and treatment modalities such as speech language pathology consultation and diet modifications have been shown to improve outcomes in general in patients with dysphagia [16–22]. It is unclear whether these interventions may mitigate the risk of mortality in the geriatric hip fracture population with dysphagia. Because of the large population of geriatric hip fracture patients, if interventions can be established to mitigate mortality risk, this could have a large clinical effect. This study did show a protective effect of SLP evaluation on mortality in this cohort of patients. This demonstrates an opportunity to improve care for these patients.

Dysphagia has previously been shown to be associated with mortality in this population [5]. A prior study investigated the timing and chronicity of dysphagia, and how those factors affected overall risk [5]. It was determined that patients with acute perioperative dysphagia were at highest risk. This meant that these patients had not had a diagnosis of dysphagia prior to their hip fracture and their first diagnosis was in the time immediately after surgery [5]. Notably, in that cohort, patients with chronic dysphagia had lower rates of mortality [5]. One hypothesis from this work was that patients who are taught or develop strategies to deal with their dysphagia may be able to better protect against complications related to dysphagia.

In the current study, speech therapy evaluation was found to be protective of mortality in hip fracture patients with dysphagia. There is robust literature demonstrating the impact of speech therapy within stroke and intensive care patients; however, there is scant literature on the impact of speech therapy within the hip fracture population. Dysphagia is common among stroke patients, and there is a high prevalence of malnutrition, aspiration, respiratory infections, and death [26–28]. Elmstahl et al. showed that with speech therapy involvement, there was an improvement in swallowing function and nutritional status in 60% of patients at follow-up [26]. In a study by Carnaby et al., stroke patients randomized to standard swallowing therapy had a trend toward reduction in death, institutionalization, and dependency when compared to usual care protocol following stroke [28]. High-intensity therapy was statistically associated with an increased number of patients who returned to normal diet and recovered swallowing function by 6 months [28]. Similar improvements in functional outcomes and decreased rates of mortality were observed in patients in the intensive care unit who received earlier interventions (within 24 h) from speech language pathology following long-term intubation [29]. Notably, our results demonstrate that SLP evaluation decreases mortality risk by nearly half with an odds ratio of 0.44 (95% CI 0.21–0.96). This supports the importance of active intervention for these patients when dysphagia is identified.

Interestingly, a modified ‘dysphagia diet’ did not have a protective effect in this cohort of patients. At the institution where the study was completed, a ‘dysphagia diet’ is different from a regular diet in that there is an option for bite sized, ground, or pureed diet compared to a diet with no restrictions on food type or size. These changes alone did not impact mortality risk. While the goal of a dysphagia diet is to minimize aspiration events and maximize nutritional intake, there was no significant difference in mortality rates between patients on a dysphagia diet and those not following such a diet within this study cohort. This suggests that dietary modifications may be important for management of dysphagia; however, diet alone may not significantly impact mortality rates.

There are several limitations of the current study. The retrospective study design limits the data to what was available in the medical record. While efforts were made to be comprehensive with the review and identifying patients with dysphagia, it is possible that it remained underreported. Due to the variability of intervention from the speech pathology team, specific interventions were not evaluated for effectiveness, only the presence of an evaluation. Furthermore, adherence to the ‘dysphagia diet’ was not measured, and just because it was ordered does not mean that patients were limiting themselves to the food provided to them by the hospital.

The findings of this study suggest routine SLP evaluations in patients with dysphagia may limit mortality in a way that diet modification alone may not. Consideration should be given to including speech language pathologists in the preoperative risk assessment or routinely involve speech language pathologists in the postoperative evaluation and care of these patients, similar to the utilization of physical and occupational therapy. Such changes could lead to more consistent evaluation for dysphagia and standardized treatment protocols including swallowing therapy. Future research should focus on longitudinal studies to explore the outcomes of different dysphagia identification and management strategies and the potential benefits of a multidisciplinary approach, combining nutritional, rehabilitative, and pharmacological interventions.

## Conclusion

This study found that SLP evaluations, in this cohort, reduced mortality in elderly hip fracture patients with a diagnosis of dysphagia. Early identification of patients with dysphagia and intervention by an SLP may improve post-operative outcomes in the elderly population. Further studies are needed to explore the full potential of SLP interventions and refine strategies for postoperative dysphagia management in elderly patients with hip fractures.

## Data Availability

I can provide the data to anyone who requests it.
